# Supplementary material for: Single-Cell Transcriptomic Analysis Reveals the Molecular Profile of Go-Opsin Photoreceptor Cells in Sea Urchin Larvae
Source: Cells. 2023 Aug 23;12(17):2134. doi: 10.3390/cells12172134 (PMC10486798; doi:10.3390/cells12172134)
Supplement: Supplementary file 1 [file cells-12-02134-s001.zip › cells-2562127-supplementary.pdf]

Single-cell transcriptomic analysis reveals the molecular profile of Go-Opsin photoreceptor cells in sea urchin larvae

Maria Cocurullo†, Periklis Paganos†, Rossella Annunziata, Danila Voronov and Maria Ina Arnone,*

Table S1 Primers used to clone the gene of interests

Gene name	Primer sequence	
Sp-Opsin3.2	Sp- Opsin3.2F	CGCCCTCTACCTGACCTTAG
	Sp- Opsin3.2R	ATTTAGGTGACACTATAGGCGCGAAAACTCTGCTGATA
Sp-Trh	Sp-TrhF	Clones gifted by Oliveri's lab (UCL)
	Sp-TrhR	Clones gifted by Oliveri's lab (UCL)

Supplementary S1 Single-cell RNA sequencing statistical data analysis

The statistical analysis of the Single-cell RNA sequencing was performed according to the Seurat scRNA-seq R package documentation [48,49].Genes that are transcribed in less than three cells and cells that have less than a minimum of 250 transcribed genes were excluded from the analysis. Datasets were normalized and variable genes were found using the Variance stabilizing transfer (VST) method with a maximum of 2000 variable features. Data integration was performed via identification of anchors between the two different objects. Nearest Neighbor (SNN) graph was com puted with 36 dimensions (resolution 1.0) to identify the clusters. Uniform Manifold Approximate and Projection (UMAP) was used to perform clustering dimensionality reduction. Cluster markers were found using the genes that are detected in at least 0.01 fraction of min.pct cells in the two clusters. The 3 dpf scRNA-seq [40,50] and 5 dpf datasets were merged using the harmony package (v0.1.0._and 30 dimensions) [51] Transcripts of all genes per cell type were identified by converting a Seurat DotPlot with all these transcripts as features into a table (ggplot2 3.2.0 R package). Subsetting analysis was performed by selecting all the cells present in neurons 4 and immune cells that express *Sp-Opsin3.2* and *Sp-Opsin2* respectively, through the Subset function incorporated into the Seurat R package.
